# Impact of varicocelectomy prior ICSI on clinical and neonatal outcomes: A multilevel analysis

**DOI:** 10.1080/20905998.2025.2550137

**Published:** 2025-08-27

**Authors:** Salah Elbashir, Ayman Rashed, Ahmed M. Fathy, Hosam Abu El-Nasr, Tamer Diab

**Affiliations:** a Department of Urology, Faculty of Medicine, Benha University, Benha, Egypt; b Department of Urology, Faculty of Medicine, 6th of October University, 6th of October, Egypt; c Department of Obstetrics and Gynecology, Faculty of Medicine, Kafrelsheikh University, Kafrelsheikh, Egypt

**Keywords:** Varicocele, varicocelectomy, male factor infertility, ICSI, ART

## Abstract

**Purpose:**

Little is known about the effectiveness and safety of varicocele repair in patients with male factor infertility undergoing ICSI treatment. Data concerning neonatal outcomes is lacking. We aimed to investigate the effect of varicocele repair prior ICSI.

**Methods:**

We analyzed retrospective data from June 2016 to January 2024 using propensity score regression analyses. We compared embryological, clinical and neonatal outcomes. We also evaluated the effect of cofounders on the rate of live birth using multivariate analysis.

**Results:**

Data from 582 ICSI cycles showed no association between livebirth and varicocele repair for male-factor patients undergoing ICSI (aOR 0.81[95% CI0.52, 1.25]; *p* = 0.3) in the adjusted multilevel logistic analysis. Four hundred and four ICSI cycles were included in the propensity score regression analysis, which confirmed the same non-significant increase of livebirth and other embryological and clinical outcomes. Data about neonatal outcomes were similar between the two groups, except for the rate of very preterm birth were higher in the varicocelectomy group compared to the untreated varicocele group.

**Conclusion:**

In male-factor infertility associated with varicocele and undergoing ICSI treatment, varicocelectomy does not significantly increase the odds of live birth compared with untreated varicocele. The increased number of very preterm birth in the varicocelectomy group may be related to the high number of multiple gestations exists in the varicocelectomy group and may not correlate to the procedures itself. Further well-powered prospective trails remain warranted.

## TWEETABLE ABSTRACT

Varicocele repair prior ICSI does not improve the overall success.

## Introduction

Varicocele, the abnormal dilation of the pampiniform plexus draining the testicle, affects about half of men with primary infertility [1,2]. Varicocele also presents in 80% of infertile men with secondary infertility. Varicocele may adversely affect and testosterone production and testicular function, leading to detrimental impact on semen parameters and sperm function [3]. Different types of varicocele repair approach have been proposed including, percutaneous embolization and varicocele ligation (e.g: subinguinal, inguinal, or retroperitoneal and laparoscopic ligations) [4].

The relationship between varicocele and male infertility remains a point of dispute [5]. For male-factor infertile patients undergoing assisted reproductive technology (ART), the situation is more controversial. Some authors claimed that varicocele compromise ART outcomes and recommended repair of varicocele to improve ART outcomes [6,7]. However, other data failed to detect this effect [8]. Several methodological limitations exist that may influence interpretations of these findings. These limitations include small sample size, inconsistences in varicocele definitions and diagnosis, variation in the treatment among studies, and retrospective nature of the studies without considering different cofounders in the statistical analysis.

To date, the clinical value of varicocelectomy prior to ART has been so far insufficient. Moreover, data linking varicocelectomy to neonatal outcomes after ICSI is lacking. This study therefore aims to retrospectively investigate whether varicocelectomy before ART affects clinical and neonatal outcomes.

## Materials and methods

### Study design and population

A retrospective cohort study was conducted in a private Center. Data of five hundreds and eighty-two couples underwent ICSI cycles during June 2016 to January 2024 were extracted from an electronic medical record system and were further analyzed. Ethics approval and protocol were approved by the scientific research ethics committee (reference number KFSIRB200–215). Informed patient consent was not deemed necessary as the data was collected routinely and anonymized for analysis.

We included male-factor patients diagnosed with clinical varicocele. Patients were included only if varicoceles were identified by palpation, Doppler duplex ultrasound and abnormal semen analysis. In our center, varicocelectomy only is performed to patients with clinical varicocele and abnormal semen analysis. Thus, inclusion of patients who underwent varicocelectomy in this study depended upon abnormal semen before varicocele repair. Patients underwent subinguinal microscope-assisted varicocelectomy were considered intervention arm, regardless of their semen analysis parameters on the day of ICSI. Patients who didn’t undergo varicocelectomy served as control.

Women were eligible if they were 18–42 years, had a body mass index (BMI) between 19 and 33 kg/m^2^ and underwent embryo transfer with blastocysts. Men were eligible if they were 18–50 years; and able to produce freshly ejaculated sperm for the treatment cycle after 3–5 days of sexual abstinence.

We excluded all cycles where pre-implantation genetic testing was performed or cycles of intracytoplasmic morphologic sperm injection (IMSI) or physiological intracytoplasmic sperm injection (PICSI). Men with azoospermia, or exposed to irradiation or chemotherapy were excluded. Women with recurrent implantation failure, recurrent pregnancy loss, uterine abnormalities or any medical conditions were excluded.

### Subinguinal microscope-assisted varicocelectomy

Subinguinal microscope-assisted varicocelectomy is routinely used for varicocele repair in our center. After anesthesia, the patient was placed in a supine position followed by applying conventional disinfection and drape surgical towels. Incision was done transversely with a length of approximately 2.5 to 3 cm at the level of an external inguinal ring. After retracting the subingunal fat, the cord was identified. The cord was dissected and freed, proximally and distally. Looping of the cord was done with a vascular tape placed underneath the cord for retraction. Any engorged veins present in the tissues outside the cord were ligated. External spermatic fascia was opened to identify the cord contents, after that dissection is assisted with a microscope with × 10–15 magnification. After identification of lymphatic vessels and arteries, sparing was done. The engorged internal spermatic veins and cremasteric vein gently dissected and isolated. We ligated each vein and then cutting in between them.

### Ovarian stimulation and ICSI

Ovarian hyperstimulation protocol was selected according to individual patient characteristics and clinician preference. The dose of gonadotropin was adjusted according to patient parameters and response. When two or more oocyte follicles reached 18 mm, triggering was achieved using hCG or gonadotrophin releasing hormone agonist, depending on the protocol used for stimulation. Transvaginal oocyte aspiration was performed 36 hours after trigger using ultrasound guidance.

Fresh ejaculates were processed using a density gradient centrifugation (SpermGrad™; Vitrolife). Intracytoplasmic sperm injection (ICSI) of only mature oocytes (MII) was performed immediately after 2–4 hours of oocytes preincubation and denudation.

### Embryo assessment and transfer

Embryos were graded according to the 2011 Istanbul consensus of the Alpha Scientists in Reproductive Medicine and ESHRE Special Interest Group of Embryology [9]. We vitrified and thawed blastocysts according to our previously published protocol [10]. Blastocysts were transferred, either fresh or frozen, under ultrasound guidance.

### Outcomes

We calculated the clinical outcomes according to reporting guidelines [11]. The primary outcome of this study is a live birth >20 weeks of gestation. A fetus death after 20 weeks of gestational age was considered stillbirth. Secondary outcomes are biochemical pregnancy (positive β-hCG value 2 weeks after ET), clinical and ongoing pregnancies (confirmed when a gestational sac with fetal heart beat by ultrasound after 4 and 12 weeks of pregnancy, respectively), miscarriage (loss of fetal cardiac activity ≤20th weeks of gestational age), implantation rate (number of gestational sacs with fetal cardiac activity divided by number of embryos transferred).

We analyzed neonatal outcomes including birth weight, preterm birth, small for gestational age, large for gestational age, appropriate for gestational age, congenital anomalies, and admission to neonatal intensive care unit (NICU). Preterm birth and very preterm birth defined as delivery before 37 and 32 weeks of gestation, respectively. Birth weight was calculated within 24 hours of birth and categorized it into low birth weight ( < 2,500 g), very low birth weight ( < 1,500 g.), and macrosomia ( > 4,000 g). We calculated major congenital anomaly, defined as structural or functional malformations identified either prenatally or at birth.

#### Statistical analysis

We analyzed all data using R package [12]. We presented descriptive data as percentages for categorical variables and medians for continuous variables. We used Chi-squared tests (χ^2^) to compare categorical variables and Wilcoxon test to compare continuous variables.

We used propensity score-match to achieve comparable baseline characteristics [13]. We considered potential confounding factors including, female and male ages, female and male body mass index (BMI), duration of infertility, varicocele laterality and grade, basal FSH, Antral follicle count (AFC), dose of gonadotropin, days of stimulation, E2 levels, number of oocytes retrieved, number of MII oocytes injected and number of embryos transferred. Cycles with missed confounding factors were excluded in the propensity score-match analysis. We further performed multivariate logistic regression analysis for the original unmatched cohort to assess the association between the primary outcome and all other variables. *P*-values <0.05 were considered statistically significant.

## Results

**Figure 1.**
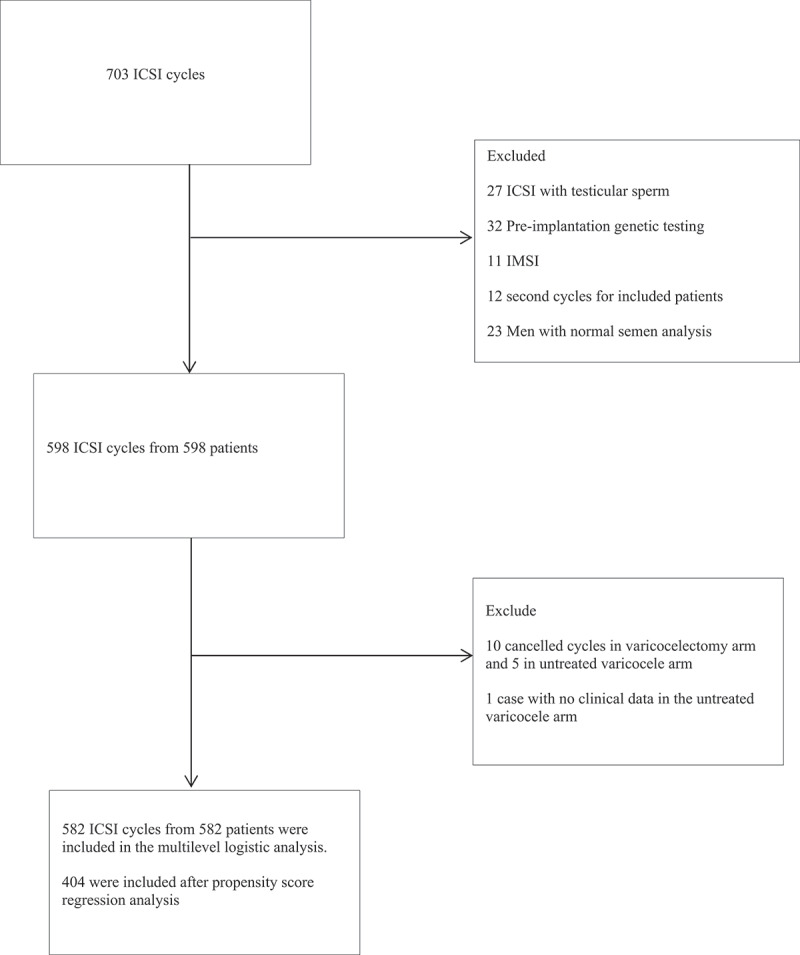
Flow chart of patient inclusion in the study.

We analyzed 582 ICSI cycles from 582 different patients in the multivariate analysis. Three hundred and fifty-nine patients underwent ICSI after varicocelectomy and 223 patients underwent ICSI with untreated clinical varicocele (Figure 1).

Table 1 provides the demographics of the two groups. Before matching, female patients whose partners underwent varicocelectomy prior ICSI were significantly older, with higher basal follicle-stimulating hormone level (FSH) and higher duration of infertility compared to patients in the control group, respectively. Sperm concentration, motility and progressive motility were significantly higher in the varicocelectomy group compared to the control group. The remaining variables were comparable between groups (Table 1). A total of 404 cycles- 202 men underwent varicocelectomy before ICSI matched 1:1 with 202 men with varicocele were included in the propensity score analysis. Before and after propensity score matching, embryological (Supplementary Table S1) and clinical outcomes (Table 2) were comparable between both groups.

**Table 1.** Baseline characteristics before and after propensity score matching.

Characteristic	Unmatched			PS matched		
	Varicocelectomy *N* = 359^*1*^	Control *N* = 223^*1*^	p-value^*2*^	Varicocelectomy *N* = 202^*1*^	Control *N* = 202^*1*^	p-value^*2*^
**Husband age (years)**	32 (29, 35)	32 (29, 35)	0.8	31 (28, 34)	31 (29, 35)	0.6
**Wife age (years)**	30 (26, 33)	29 (25, 32)	**0.042**	29 (24, 32)	29 (24, 31)	0.4
**Husband BMI**	27.17 (25.90, 29.07)	26.99 (25.66, 29.02)	0.3	27.57 (26.04, 29.56)	27.21 (25.66, 29.40)	0.2
Unknown	2	0		2	0	
**Female BMI**	26.8 (24.8, 29.7)	26.9 (25.0, 29.3)	> 0.9	27.1 (25.0, 30.1)	26.9 (25.2, 29.4)	0.6
**Duration of infertility (years)**	3 [3,4]	3 [2,4]	**0.004**	3 [2,4]	3 [2,4]	0.8
Unknown	1	0				
**Laterality**			0.5			0.8
Unilateral	320 (90%)	195 (88%)		178 (89%)	176 (88%)	
Bilateral	36 (10%)	26 (12%)		23 (11%)	25 (12%)	
Unknown	3	2		1	1	
**Varicocele grade**			0.2			0.3
II	160 (45%)	84 (39%)		87 (44%)	77 (39%)	
I	97 (27%)	75 (34%)		51 (26%)	65 (33%)	
III	98 (28%)	59 (27%)		62 (31%)	58 (29%)	
Unknown	4	5		2	2	
**Semen volume (mL)**	3.00 (2.50, 3.50)	3.00 (2.00, 4.00)	0.5	3.00 (2.50, 3.50)	3.00 (2.00, 4.00)	0.5
**Sperm concentration (million/mL)**	14 [8,14]	11 [7,15]	** < 0.001**	14 [8,14]	11 [7,15]	**0.004**
**Sperm progressive motility (%)**	15 [10,16]	10 [5,10]	** < 0.001**	15 [10,17]	10 [5,10]	** < 0.001**
**Sperm total motility (%)**	45 (35, 55)	40 [17, 55]	** < 0.001**	48 (35, 55)	35 [17, 50]	** < 0.001**
**Sperm normal form (%)**	1 [1,2]	1 (0, 2)	0.8	1 [1,2]	1 (0, 3)	0.6
**Basal FSH level (mIU/mL)**	6.40 (6.10, 6.70)	6.30 (5.90, 6.50)	** < 0.001**	6.30 (5.90, 6.60)	6.30 (5.90, 6.50)	0.5
Unknown	24	21				
**Antral follicle count**	17 [15,17]	17 [15,18]	0.9	17 [13,17]	17 [15,18]	0.4
Unknown	22	24		1	3	
**Total dose of gonadotropins (IU)**	2,475 (2,100, 2,925)	2,475 (2,175, 2,925)	0.7	2,475 (2,100, 2,850)	2,475 (2,175, 2,925)	0.6
Unknown	25	25		2	5	
**Days of stimulation**	11 [10,11]	11 [10,11]	0.8	11 [10,11]	11 [10,11]	0.8
Unknown	23	24		2	4	
**Estradiol level (pg/mL)**	2,800 (2,100, 3,500)	2,755 (2,120, 3,400)	0.9	2,750 (1,980, 3,450)	2,780 (2,200, 3,400)	0.4
Unknown	25	21		3	1	

^*1*^Median (Q1, Q3); n (%).

^*2*^Wilcoxon rank sum test; Pearson’s Chi-squared test.

**Table 2.** Clinical outcomes of varicocelectomy group and control group before and after propensity score matching.

Characteristic	Unmatched			PS matched		
	Varicocelectomy *N* = 349^*1*^	Control *N* = 217^*1*^	p-value^*2*^	Varicocelectomy *N* = 201^*1*^	Control *N* = 198^*1*^	p-value^*2*^
Transferred embryos	2 [2]	2 [2]	> 0.9	2 [2]	2 [2]	> 0.9
**Biochemical pregnancy rate**	213 (61%)	126 (58%)	0.5	122 (61%)	115 (58%)	0.6
**Implanted embryos**	1 (0, 1)	1 (0, 1)	0.5	1 (0, 1)	1 (0, 1)	0.7
Unknown	0	1		0	1	
**Clinical pregnancy rate**	201 (58%)	119 (55%)	0.6	115 (57%)	109 (55%)	0.7
Unknown	0	1		0	1	
**Ongoing pregnancy rate**	184 (53%)	106 (50%)	0.4	109 (54%)	97 (49%)	0.3
Unknown	2	3		0	1	
**Stillbirth rate**	2 (0.6%)	1 (0.5%)	> 0.9	1 (0.5%)	1 (0.5%)	> 0.9
Unknown	5	4		2	2	
**Livebirth rate**	168 (49%)	98 (46%)	0.5	100 (49.7%)	90 (46%)	0.4
Unknown	5	4		2	2	
**Singleton livebirth rate**	112 (33%)	69 (32%)	> 0.9	66 (33%)	64 (33%)	> 0.9
Unknown	5	4		2	2	
**Multiple livebirth rate**	56 (16%)	29 (14%)	0.4	34 (17%)	26 (13%)	0.3
Unknown	5	4		2	2	

^*1*^n (%); Median (Q1, Q3).

^*2*^Pearson’s Chi-squared test; Wilcoxon rank sum test; Fisher’s exact test.

The likelihood of adverse neonatal outcomes did not differ between men with treated and untreated varicocele (Table 3). Although the mean gestational age was similar between both groups, the rate of very preterm birth was higher in the varicocelectomy group compared to untreated varicocele group (Table 3).

**Table 3.** Neonatal outcomes of varicocelectomy group and control group before and after propensity score matching.

Characteristic	Unmatched			PS matched		
	Varicocelectomy *N* = 225^*1*^	Control *N* = 125^*1*^	p-value^*2*^	Varicocelectomy *N* = 134^*1*^	Control *N* = 115^*1*^	p-value^*2*^
**Congenital anomalies (%)**	0 (0%)	2 (1.6%)	0.13	0 (0%)	2 (1.7%)	0.2
**NICU admission rate**	25 (11%)	12 (9.6%)	0.7	17 (13%)	8 (7.0%)	0.13
**Gestational age at birth (weeks)**	38 (36, 39)	38 (37, 39)	0.068	38 (36, 39)	38 (37, 39)	0.085
Unknown	0	1		0	0	
**Gestational age category**			**0.020**			**0.014**
Term birth	160 (71%)	101 (81%)		93 (69%)	93 (81%)	0.04
Preterm birth	57 (25%)	23 (19%)		34 (25%)	22 (19%)	0.25
Very preterm birth	8 (3.6%)	0 (0%)		7 (5.2%)	0 (0%)	0.01
Unknown	0	1				
**Birthweight**	2,900 (2,700, 3,200)	3,000 (2,700, 3,350)	**0.037**	2,900 (2,700, 3,200)	3,000 (2,700, 3,400)	0.05
Unknown	1	1		1	0	
**Birthweight category**			0.5			0.4
Marcosomia	3 (1.3%)	3 (2.4%)		2 (1.5%)	3 (2.6%)	
Normal birthweight	189 (84%)	110 (88%)		112 (84%)	101 (88%)	
Low birthweight	31 (14%)	12 (9.6%)		18 (13%)	11 (9.6%)	
Very low birthweight	2 (0.9%)	0 (0%)		2 (1.5%)	0 (0%)	

^*1*^n (%); Median (Q1, Q3).

^*2*^Fisher’s exact test; Pearson’s Chi-squared test; Wilcoxon rank sum test.

Estimates of multivariate analysis are presented in the supplementary table S2. In the adjusted multivariate regression model, only associations with male age (aOR 1.59 [95% CI 1.06–2.44]; *p* = 0.028), presence of grade 3 of varicocele (aOR 0.46 [95% CI 0.28–0.76]; *p* = 0.003), sperm concentration (aOR 0.81 [95% CI 0.66–1.00]; *p* = 0.048), and top-quality blastocysts (aOR 2.46 [95% CI1.77–3.47]; *p*= < 0.001) were significant.

## Discussion

### Main finding

In male-factor infertility patients diagnosed with varicocele and underwent ICSI, we found no difference in the rate of live birth compared to untreated varicocele in both multilevel and propensity score regression analyses. Our data showed no association between repairing of varicocele prior ICSI with embryological and clinical outcome measures. In addition, varicocelectomy prior ICSI did not affect the risk of adverse neonatal outcomes.

### Interpretation of findings

Our findings are constant with one large cohort study [14]. However, they are contrary to other reports in the literature suggesting that varicocelectomy could improve the ART outcomes [7,15]. A meta-analysis by Esteves et al., 2016 that included four retrospective studies with 870 ICSI cycles, found improved rates of clinical pregnancy and live birth in the varicocelectomy group compared to control untreated varicocele group underwent ICSI [6]. Another meta-analysis by Kirby et al., 2016 showed a significant increase in the rate of live birth after varicocelectomy for men with oligospermia compared to untreated control [16]. A more recent meta-analysis by Teng et al., 2025 found a statistical increase in the rate of clinical pregnancies in ICSI patients after varicocelectomy compared to the control group [15]. In contrast, our data failed to find such correlation for the clinical outcomes after ICSI. Some of these differences across the literature interpretation might be due to the methodological limitations with confounding bias. In addition, patient population and characteristics differ significantly across the studies. Unlike existing studies in the literature, we considered confounding bias using both propensity score matching and multilevel analyses.

A potential explanation for why no association between varicocele repair and ART success was detected may be the DNA repair capacity of the oocytes of the anticipated sperm DNA damage associated with varicocele [17]. Moreover, the use of ICSI in cases of male infertility may also be beneficial to skip any functional alternation of the sperm [18] that is induced by the presence of testicular varicoceles.

Concerns about the safety of the ICSI procedures persist [19]. Some reports showed that ICSI may carry risks of adverse effect on intrauterine growth and offspring [20]. This risk may be due to paternal DNA alternation or abnormal sperm. Some authors suggested the use of interventions and targeted therapies that could reduce oxidative stress and sperm DNA damage could be beneficial to achieve ICSI as safe as possible [18,20]. Although varicocele repair has been suggested for this purpose, our data showed no effect on intrauterine growth as compared to untreated varicocele. In regard to the increased number very preterm birth, this may be correlated to the relatively high number of multiple gestations exists in the varicocelectomy group and may not correlate to the procedures itself.

In our practice, we use microsurgical varicocelectomy for varicocele repair. The current available evidence recommends microsurgical varicocelectomy for the treatment of varicocele because it is associated with higher spontaneous pregnancy and reduced postoperative complications as compared to other techniques [21,22]. However, no such comparisons between other techniques in infertile men undergoing ART are available. Given the potential risks and additional cost associated with varicocele repair procedures, with no improve in success rates after ICSI, it seems unnecessary to perform such a surgery for infertile men undergoing ICSI. Additional large cohort prospective studies and RCTs remain warranted to prove our findings.

### Strengths and limitations

Our study poses several strengths. First, our study inevitably contributes to the literature, as previous reports on the effect of treated and untreated varicocele on neonates from ICSI cycles are lacking. Second, this is the largest report to date with detailed aforementioned outcomes. Third, although previous studies have investigated the effect of varicocele with and without repair on the overall success rate of ICSI, these studies have not considered the effect of cofounders. Our analyses expand on this body of literature by considering all the confounding variables that could affect the interpretation of the results.

Our study poses several limitations. Retrospective analyses pose a series of methodological limitations including, lack of randomization and confounding effect biases. To overcome these limitations in the current study, we adjust for the potential confounders using propensity score regression, an analysis that determines each patient’s propensity to match treated with untreated subjects. We also applied multivariate regression analysis to address variables that affects live birth. Our study was restricted to microsurgical varicocelectomy at a single center. Although this could undermine the generalizability of our finding, it controls for potential differences in clinical practices and treatment between fertility centers. A further limitation of our study is that the duration of follow-up after varicocelectomy is not determined, which is considered a prominent confounder. Future studies might show how the type of varicocele repair or surgery, the effect of grade of varicocele, the duration between varicocelectomy and ICSI and the severity of semen profile and geographical location that may convey benefit. Clinical and neonatal outcomes measures need to be carefully investigated in these patients in future prospective trails. More information is also required to validate the effects of varicocelectomy before ICSI on longer-term outcomes.

## Conclusions

We found no association of clinical varicocele with adverse embryological, clinical or perinatal outcomes after ICSI, compared to varicocele-repaired patients. Given the potential risk and additional cost of varicocelectomy, this information is useful in counseling patients with clinical varicocele and undergoing ART program. Further information from RCTs is needed to ascertain our findings.

## Supplementary Material

Supplemental Material

Supplemental Material

## Data Availability

All data can be provided by the author, upon reasonable request.
