# Supplementary material for: Impact of varicocelectomy prior ICSI on clinical and neonatal outcomes: A multilevel analysis
Source: Arab J Urol. 2025 Aug 27;24(2):114–20. doi: 10.1080/20905998.2025.2550137 (PMC13045194; doi:10.1080/20905998.2025.2550137)
Supplement: Supplemental Material [file TAJU_A_2550137_SM2826.docx]

| **Supplementary table 2:** Multivariate regression model for potential predictors of higher livebirth rate | | | | | | |
| --- | --- | --- | --- | --- | --- | --- |
| **Characteristic** | **Unmatched** | | | **PS matched** | | |
|  | **OR***^1^* | **95% CI***^1^* | **p-value** | **OR***^1^* | **95% CI***^1^* | **p-value** |
| **Arm** |  |  |  |  |  |  |
| Varicocelectomy | — | — |  | — | — |  |
| Control | 0.81 | 0.52, 1.25 | 0.3 | 0.63 | 0.38, 1.02 | 0.064 |
| **Husband age (years)** | 1.59 | 1.06, 2.44 | **0.028** | 1.79 | 1.12, 2.93 | **0.018** |
| **Husband BMI** | 1.00 | 0.83, 1.22 | >0.9 | 0.96 | 0.75, 1.21 | 0.7 |
| **Wife age (years)** | 0.70 | 0.45, 1.07 | 0.10 | 0.64 | 0.38, 1.06 | 0.086 |
| **Female BMI** | 0.93 | 0.76, 1.13 | 0.4 | 1.02 | 0.81, 1.29 | 0.9 |
| **Duration of infertility (years)** | 0.96 | 0.77, 1.20 | 0.7 | 1.01 | 0.78, 1.33 | >0.9 |
| **Varicocele laterality** |  |  |  |  |  |  |
| Bilateral | — | — |  | — | — |  |
| Unilateral | 1.13 | 0.62, 2.08 | 0.7 | 1.00 | 0.50, 2.00 | >0.9 |
| **Varicocele grade** |  |  |  |  |  |  |
| I | — | — |  | — | — |  |
| II | 0.78 | 0.49, 1.23 | 0.3 | 0.90 | 0.51, 1.57 | 0.7 |
| III | 0.46 | 0.28, 0.76 | **0.003** | 0.52 | 0.29, 0.95 | **0.033** |
| **Sperm concentration (million/ mL)** | 0.81 | 0.66, 1.00 | **0.048** | 0.70 | 0.54, 0.89 | **0.005** |
| **Sperm progressive motility (%)** | 1.07 | 0.86, 1.33 | 0.6 | 0.99 | 0.77, 1.28 | >0.9 |
| **Sperm normal form (%)** | 0.96 | 0.78, 1.18 | 0.7 | 0.84 | 0.66, 1.07 | 0.2 |
| **Basal FSH level (mIU/ mL)** | 1.13 | 0.93, 1.37 | 0.2 | 1.11 | 0.88, 1.40 | 0.4 |
| **Total dose of gonadotropins (IU)** | 0.99 | 0.81, 1.21 | >0.9 | 1.03 | 0.82, 1.31 | 0.8 |
| **Days of stimulation** | 1.04 | 0.86, 1.27 | 0.7 | 1.07 | 0.85, 1.35 | 0.6 |
| **Estradiol level (pg/ mL)** | 0.79 | 0.53, 1.15 | 0.2 | 0.71 | 0.44, 1.09 | 0.12 |
| **Antral follicle count** | 1.22 | 0.93, 1.61 | 0.2 | 1.14 | 0.82, 1.59 | 0.4 |
| **Cumulus-oocyte complex** | 1.52 | 0.65, 3.60 | 0.3 | 1.69 | 0.61, 4.71 | 0.3 |
| **Metaphase II oocytes** | 0.50 | 0.20, 1.24 | 0.13 | 0.54 | 0.18, 1.67 | 0.3 |
| **Top-quality day 3 embryos** | 1.03 | 0.69, 1.54 | 0.9 | 0.79 | 0.48, 1.30 | 0.4 |
| **Top-quality blastocyst** | 2.46 | 1.77, 3.47 | **<0.001** | 3.13 | 2.07, 4.87 | **<0.001** |
| **Transferred embryos** | 1.24 | 0.99, 1.58 | 0.074 | 1.15 | 0.89, 1.50 | 0.3 |
| *^1^* aOR = Adjusted Odds Ratio, CI = Confidence Interval | | | | | | |
