# Supplementary material for: Impact of varicocelectomy prior ICSI on clinical and neonatal outcomes: A multilevel analysis
Source: Arab J Urol. 2025 Aug 27;24(2):114–20. doi: 10.1080/20905998.2025.2550137 (PMC13045194; doi:10.1080/20905998.2025.2550137)
Supplement: Supplemental Material [file TAJU_A_2550137_SM2820.docx]

| **Supplementary table 1:** Embryological outcomes of varicocelectomy group and control group before and after propensity score matching | | | | | | |
| --- | --- | --- | --- | --- | --- | --- |
| **Characteristic** | **Unmatched** | | | **PS matched** | | |
|  | **Varicocelectomy** N = 359*^1^* | **Control** N = 223*^1^* | **p-value***^2^* | **Varicocelectomy** N = 202*^1^* | **Control** N = 202*^1^* | **p-value***^2^* |
| **Cumulus-oocyte complex** | 15 (12, 20) | 16 (12, 21) | 0.6 | 15 (12, 20) | 16 (12, 21) | 0.4 |
| **Metaphase II oocytes** | 13 (10, 17) | 13 (10, 18) | 0.6 | 13 (10, 16) | 14 (10, 18) | 0.3 |
| **Fertilized oocytes** | 10 (7, 13) | 10 (8, 14) | 0.7 | 10 (8, 13) | 11 (8, 14) | 0.4 |
| **Cleaved embryos** | 10 (7, 13) | 10 (8, 13) | 0.7 | 10 (7, 13) | 10 (8, 13) | 0.4 |
| **Top-quality day 3 embryos** | 6 (4, 8) | 6 (4, 8) | 0.6 | 6 (4, 8) | 6 (4, 8) | >0.9 |
| **Blastocysts** | 6 (4, 8) | 6 (4, 8) | 0.5 | 5 (4, 8) | 6 (4, 8) | 0.4 |
| **Top-quality blastocysts** | 4 (2, 5) | 4 (2, 5) | 0.4 | 4 (2, 5) | 4 (2, 5) | 0.5 |
| *^1^* Median (Q1, Q3)  *^2^* Wilcoxon rank sum test | | | | | | |
